# Dysbiosis of Gut Microbiome Aggravated Male Infertility in Captivity of Plateau Pika

**DOI:** 10.3390/biom14040403

**Published:** 2024-03-26

**Authors:** Liangzhi Zhang, Xianjiang Tang, Chao Fan, Shi’en Ren, Qi Cheng, Huakun Zhou, Kai Liu, Shangang Jia, Yanming Zhang

**Affiliations:** 1 Key Laboratory of Adaptation and Evolution of Plateau Biota, Northwest Institute of Plateau Biology, Chinese Academy of Sciences, Xining 810008, China; lzzhang@nwipb.cas.cn (L.Z.); tangxianjiang@nwipb.cas.cn (X.T.); fanchao@nwipb.cas.cn (C.F.); renshien@nwipb.cas.cn (S.R.); chengqi@nwipb.cas.cn (Q.C.); 2 Key Laboratory of Restoration Ecology of Cold Area in Qinghai Province, Northwest Institute of Plateau Biology, Chinese Academy of Sciences, Xining 810008, China; hkzhou@nwipb.cas.cn; 3 Qinghai Provincial Grassland Station, Xining 810008, China; 17309789990@163.com; 4 College of Grassland Science and Technology, China Agricultural University, Beijing 100193, China

**Keywords:** wild animals, captivity, gut microbiota, short-chain fatty acid, male infertility

## Abstract

Captivity is an important and efficient technique for rescuing endangered species. However, it induces infertility, and the underlying mechanism remains obscure. This study used the plateau pika (*Ochotona curzoniae*) as a model to integrate physiological, metagenomic, metabolomic, and transcriptome analyses and explore whether dysbiosis of the gut microbiota induced by artificial food exacerbates infertility in captive wild animals. Results revealed that captivity significantly decreased testosterone levels and the testicle weight/body weight ratio. RNA sequencing revealed abnormal gene expression profiles in the testicles of captive animals. The microbial α-diversity and Firmicutes/Bacteroidetes ratio were drastically decreased in the captivity group. *Bacteroidetes* and *Muribaculaceae* abundance notably increased in captive pikas. Metagenomic analysis revealed that the alteration of flora increased the capacity for carbohydrate degradation in captivity. The levels of microbe metabolites’ short-chain fatty acids (SCFAs) were significantly high in the captive group. Increasing SCFAs influenced the immune response of captivity plateau pikas; pro-inflammatory cytokines were upregulated in captivity. The inflammation ultimately contributed to male infertility. In addition, a positive correlation was observed between *Gastranaerophilales* family abundance and testosterone concentration. Our results provide evidence for the interactions between artificial food, the gut microbiota, and male infertility in pikas and benefit the application of gut microbiota interference in threatened and endangered species.

## 1. Introduction

Captive breeding is one of the most effective technologies for protecting endangered wild animals concerning species conservation [1]. Wild animals that are introduced into captivity must endure drastic changes imposed by artificial conditions, such as space limitation, unnatural housing structure, and diet, which eventually affects their health, nutrition, physiology, and immune system [2]. Evidence from amphibians, birds, and mammals suggests that captive breeding conditions can disrupt the composition and structure of the gut microbiota [3,4,5]. This disruption may reduce microbial diversity, followed by function changes in the gut microbiota [6], and could also elicit metabolic [7], endocrine [8], and immune disorders [9], consequently impairing host health and reproduction. 

Artificial diets can induce microbiota dysbiosis in mice and impair their reproductive systems [10,11], as spermatogenesis defects have been observed in relation to elevated endotoxins, the dysregulation of testicular gene expression, and localized epididymal inflammation [12,13]. The gut microbiota is associated with hormone production and reproductive success in terms of the degradation of steroid hormones, such as hydroxysteroid dehydrogenases (HSDs), in wildlife [14,15]. Sex hormones may participate in the communication between microorganisms and hosts and modulate host reproduction [15,16]. Dysbiosis of the gut microbiota plays a role in the regulation of host hormone levels via close communication between the gut and testicular tissues, thereby inhibiting wild animal reproduction [14,17].

The *Ochotona* genus consists of 34 species and is tentatively divided into 5 subgenera [18,19]. Currently, there are 24 species distributed in the Qinghai–Tibet Plateau [20]. With global warming and overgrazing by livestock, approximately 90% of these species are endangered [21]. Plateau pikas (*Ochotona curzoniae*) belong to the lagomorph family Ochotonidae. They are considered keystone and umbrella species in the Qinghai–Tibetan alpine ecosystem [22,23]. They are an important indicator of ecosystem health, contribute to plant biodiversity, and play irreplaceable roles in maintaining the integrity and stability of the food structure [23,24]. Plateau pikas inhabit alpine meadows with altitudes of 3200 to 5300 m. The habitat is dominated by herbaceous perennids. Although the food preferences of plateau pikas vary in different habitats, *Oxytropis*, *Elymus nutans*, and *Poa pratensis* were preferred [25]. They are seasonally breeding small mammals. Pikas are social animals, and a family group generally consists of two to five adult members. The breeding season lasts from April to August. During breeding season, females can produce two to five litters with a 3-week interval between each litter [26]. 

However, recently, the number of plateau pikas has been dropping in meadows, and their genetic diversity has also been lost [27]. Extensive studies on plateau pikas have been conducted regarding their systematic evolution, physiological and biochemical characteristics [28], and the behavioral and seasonal characteristics of the intestinal flora [29]. However, the conservation and rescue of this species are rare. Plateau pikas are sensitive and timid, and maintaining them in captivity poses considerable challenges to their survival. Difficulties in adaptability and reproduction are major issues in captivity during the rescue and conservation of these animals.

In this study, captive plateau pikas were used as a model to explore the factors underlying their male infertility in comparison with wild populations. The study aimed to (1) determine the effect of captivity on abnormal androgen levels and testes morphology; (2) explore the influence of captivity on the structure and function of the gut microbiome and metabolites; and (3) establish the manner in which the dysbiosis of gut microbiota and metabolites closely communicates with the host and then affects host reproduction. 

## 2. Materials and Methods

### 2.1. Sample Collection

In January, 30 healthy adult females’ samples were trapped using a live-trapping method and locked in cages previously sterilized with 75% alcohol. Fresh feces were collected in 2-mL tubes (Sigma-Aldrich, St. Louis, MO, USA), immediately frozen, and stored in liquid nitrogen. Then, samples were weighted using a spring scale (±2 g, PESO-40300/7, PESOLA, Zurich, Switzerland) and transferred to the laboratory at the Northwest Plateau Institute of Biology, Chinese Academy of Sciences, Xining, Qinghai, China.

Subsequently, 8 samples were euthanized with isoflurane and dissected on a sterile dissecting table. We collected blood from the heart chambers to measure hormone levels. Blood samples were taken quietly overnight in fasting conditions. Serum was collected by centrifugation and stored at −80 °C until use. Additionally, the cecal contents, cecum, and testis were collected and cryopreserved in a portable liquid nitrogen tank. Afterwards, the other testis was weighed on an electronic balance (±0.0001 g; FA2104, Shanghai Liangping Instrument, Co., Ltd., Shanghai, China). 

The remaining 22 samples were housed in plastic cages (45 × 32 × 19 cm) with wood sawdust bedding (one individual escaped). Water and food were provided ad libitum. The captivity conditions were as follows: artificial feed (pelleted feed, rabbit maintenance feed; Beijing Keao Xieli Feed Co., Ltd., Beijing, China), natural light, ventilation by windows, and no heating (maintaining the same ambient temperature in the field during the entire captivity period). The main components of artificial feed are corn meal, bean pulp, and lucerne meal (nutrient content of the artificial feed is shown in Table S1). The pikas were fed until April of the following year (denoted as Cap), and fresh feces were collected in 2 mL tubes (Sigma-Aldrich, St. Louis, MO, USA). Then, 9 captive samples were selected, euthanized, and dissected on a sterile dissecting table, and samples were collected and stored according to the above procedure.

In April, another 34 healthy adult female samples were trapped and weighed at the same site (Wild_Apr) and locked in cages previously sterilized with 75% alcohol. Fresh feces were collected in 2 mL tubes (Sigma-Aldrich, St. Louis, MO, USA), immediately frozen, and stored in liquid nitrogen. Then, 12 samples were transferred to the laboratory, and others were released at the location of capture. Finally, 8 wild plateau pikas in April were euthanized and dissected on a sterile dissecting table, and samples were collected and stored according to the above procedure. 

In total, 85 healthy adult females’ fresh feces were used for 16S rRNA analysis (Wild_Apr group *n* = 34; Wild_Jan groups *n* = 30; Cap group *n* = 21); 27 healthy adult females’ serum were used for hormone analysis (Wild_Apr group *n* = 12; Wild_Jan groups *n* = 6; Cap group *n* = 9); 28 healthy adult females were used for testicle weight and body weight analysis (Wild_Apr group *n* = 12; Wild_Jan groups *n* = 6; Cap group *n* = 9); 29 healthy adult females’ cecal contents were used for SCFAs analysis (Wild_Apr group *n* = 12; Wild_Jan groups *n* = 8; Cap group *n* = 9); 26 healthy adult females’ cecal contents were used for metagenomic sequencing (Wild_Apr group *n* = 12; Wild_Jan groups *n* = 5; Cap group *n* = 9); 9 healthy adult females’ cecum epithelium and testes were used for mRNA sequencing (Wild_Apr group *n* = 3; Wild_Jan groups *n* = 3; Cap group *n* = 3). 

The samples used in this study are listed in Table S2. This study was approved by the Animal Ethics Committee of Northwest Plateau Institute of Biology, Chinese Academy of Sciences (NWIPB2019110801).

### 2.2. Serum Hormone Metabolite Measurement

The levels of serum dopamine, thyroxine, melatonin, cortisol, and sex hormones, including testosterone, estrone, estradiol, dehydroepiandrosterone, and stanolone, were determined using ultra-high-performance liquid chromatography–mass spectrometry (UHPLC-MS) analysis using a 5500 QTRAP mass spectrometer by Shanghai Bioprofile Technology Co., Ltd. (Shanghai, China). 

Briefly, 80 µL of serum was mixed with cold methanol/acetonitrile via vortexing and then centrifuged at 4 °C (14,000× *g*). The supernatants were dried under a vacuum for LC-MS analysis. The sample was spiked with 0.1 μg of re-standard Aldosterone-d8 as an internal standard during sample extraction. Samples were separated using a Nexera X2 LC-30AD ultra-high-pressure liquid chromatography (Shimadzu, Tokyo, Japan). Mobile phase: solution A is 10 mM ammonium acetate solution pH 8.0, and solution B is 100% acetonitrile. The samples were placed in a 4 °C autosampler with a column temperature of 40 °C, a flow rate of 300 μL/min, and an injection volume of 5 μL. The liquid phase gradient is as follows: 0–5 min, liquid B changed linearly from 20% to 65%; 5–7 min, liquid B changed linearly from 65% to 100%; 7–10 min, liquid B is maintained at 100%; 10–10.1 min, liquid B changed linearly from 100% to 20%; 10.1–13 min, liquid B was maintained at 20%. Mass spectrometric analysis was performed using a 5500 QTRAP mass spectrometer (AB SCIEX, Boston, MA, USA) in positive-ion mode. ESI source parameters: Source Temperature 550 °C, Ion Source Gas1 (GAS1): 40, Ion Source Gas2 (GAS2): 50, Curtain Gas (CUR): 35, Ion Spray Voltage Floating (ISVF) 5500 V. MRM mode was used to detect the transitions. Peak area and retention time were extracted using MultiQuant software (3.0.2). Animal hormone standards were used to adjust retention times for metabolite identification. The information on animal hormone standards is listed in Table S3. The sample concentration (μg/mL) was calculated for each sample based on the ratio of the mass spectrometry peak area of different metabolites obtained from each sample to the mass spectrometry peak area of the internal standard Aldosterone-d8. Based on the volume of the re-dissolved sample and the initial total amount of the sample, the concentrations of different metabolites in the initial sample (ng/mL) were finally obtained.

### 2.3. Extraction of Fecal Genomic

Fecal DNA was extracted from stool samples using the QIAamp DNA Stool Mini Kit (Qiagen 51504; Hilden, Germany). Briefly, the stool samples were rinsed with inhibitEX buffer and centrifuged; the supernatant was transferred to a new tube with the proteinase K and buffer AL, incubated at 70 °C for 10 min, and mixed with ethanol by vortexing; the mixture was transferred to the spin column; then, the mixture was rinsed by buffer AW1 and AW1; finally, the spin column with the buffer ATE were transferred a new tube and DNA were eluted by the membrane via centrifugation. DNA concentration was determined using a NanoDrop ND-1000 spectrophotometer (Thermo Scientific, Waltham, MA, USA).

### 2.4. The 16S rDNA Gene Amplification and Sequencing

The hyper-variable V3–V4 region of the 16S rDNA gene was amplified by PCR using primers 341F (5′-CCTAYGGGRBGCASCAG-3′) and 806R (5′-GGACTACNNGGGTATCTAAT-3′). The PCR products were purified and quantified using a QuantiFluorTM fluorometer (Promega, Madison, WI, USA). Negative controls for PCR amplification were reactions without DNA. The PCR products were purified and quantified using a fluorometer (QuantiFluor; Promega, Madison, WI, USA) and then sequenced on a HiSeq 2500 platform using the PE250 model (Novogene, Beijing, China). The qualified DNA samples were randomly sheared to a length of approximately 350 bp using a Covaris ultrasonic crusher (Woburn, MA, USA). The entire library was prepared using the following steps: end repair, adding a 3′ poly-A tail, ligating adapters, purification, and PCR amplification. Library quality was assessed on a Qubit 2.0 Fluorometer (Thermo Scientific, Waltham, MA, USA) and Agilent Bioanalyzer 2100 system (Agilent Technologies, Palo Alto, CA, USA). The library was sequenced on the Illumina HiSeq platform (San Diego, CA, USA). The sequencing produced an average of 540 million reads (approximately 12 GB) per sample. 

### 2.5. Analysis of Sequencing Data

The 16S sequences were normalized, filtered, and processed according to the protocols provided by the QIIME pipeline (version 1.9.1) [30]. We normalized each sample to an equal sequencing depth and clustered the reads into operational taxonomic units (OTUs) based on 97% identity using UPARSE version 7.1 [31]. OTU taxonomy was assigned using RDP Classifier version 2.2 against the 16S rRNA database (Release 13.5, http://greengenes.secondgenome.com/, accessed on 13 December 2019) based on a confidence threshold of 0.97 [32]. 

Metagenomic data were analyzed using the free online platform Majorbio Cloud (https://cloud.majorbio.com/page/task/index.html, accessed on 13 July 2020). The paired-end Illumina reads were trimmed, and low-quality reads (length < 50 bp, quality value < 20 or N bases) were removed using fastp (version 0.20.0, https://github.com/OpenGene/fastp, accessed on 13 July 2020) [33]. The clean reads were assembled using MEGAHIT (version 1.1.2; on https://github.com/voutcn/megahit, accessed on 13 July 2020). [34]. Contigs of >300 bp in length were selected for further gene prediction and annotation. Open reading frames (ORFs) were predicted using MetaGene [35] (http://metagene.cb.k.u-tokyo.ac.jp/, accessed on 13 July 2020). Predicted ORFs with a length of ≥100 bp were retrieved and translated into amino acid sequences using the NCBI for Biotechnology Information Translation table (http://www.ncbi.nlm.nih.gov/Taxonomy/taxonomyhome.html/index.cgi?chapter=tgencodes#SG1, accessed on 13 July 2020). A nonredundant gene set was constructed using CD-HIT (version 4.6.1; http://www.bioinformatics.org/cd-hit/, accessed on 13 July 2020) [36]. After quality control, reads were mapped onto the nonredundant gene set using the SOAP aligner (version 2.21, http://soap.genomics.org.cn/, accessed on 13 July 2020), and the gene abundance for each sample was determined [37]. The Kyoto Encyclopedia of Genes and Genomes (KEGG) annotation was performed using Diamond [38] (version 0.8.35, http://www.diamondsearch.org/index.php, accessed on 13 July 2020) against the KEGG database (http://www.genome.jp/kegg/, accessed on 13 July 2020). Carbohydrate-active enzyme annotation was performed using hmmscan (http://hmmer.janelia.org/search/hmmscan, accessed on 13 July 2020) against the CAZy database (http://www.cazy.org/, accessed on 13 July 2020).

### 2.6. Short-Chain Fatty Acids Measurement

The acetate, propionate, butyrate, isobutyric, isovaleric, valeric, and caproic acid contents were measured using gas chromatography/mass spectrometry (GC-MS) [39]. The experimental procedures were based on the previous methods [40]. Briefly, 100 mg of feces was mixed with 0.005 M aqueous NaOH containing IS (Internal standard, 5 µg/mL caproic acid-d3), and the mixture was centrifuged at 12,000 rpm at 4 °C for 10 min. The supernatant was transferred into a 15 mL centrifuge tube (BBI, Shanghai, China) with the solution (water: 300 µL, propanol 300 µL and pyridine 200 µL), and then 100 µL of propyl chloroformate (PCF) was added and vortexed for 30 min. The derivatization reaction proceeded under ultrasonication for 1 min with the hexane (300 µL). This is the first extraction. The reaction mixtures were vortexed and centrifuged. Then, 300 µL of the supernatant in the hexane layer was transferred to an autosampler vial. After that, the second extraction began with another 200 µL of hexane. A total of 500 µL of the derivatized extract was collected in the autosampler vial. The mixture was briefly vortexed prior to GC-MS analysis. Derivatized samples were separated using the Chromatographic column Agilent HP-INNOWAX capillary column (30 m × 0.25 mm × 0.25 μm, Agilent Technologies, Santa Clara, CA, USA). The chromatographic conditions were split injection, injection volume 1 μL, and split ratio 10:1. The inlet temperature is 250 °C; the ion source temperature is 230 °C; the transfer line temperature is 250 °C; the quadrupole temperature is 150 °C. The starting temperature of programmed heating is 90 °C; then, it is heated to 120 °C at 10 °C/min; to 150 °C at 5 °C/min; finally, to 250 °C at 25 °C/min for 2 min. The carrier gas was helium, and the carrier gas flow rate was 1.0 mL/min. MS conditions: electron impact ionization (EI) source, full scan and SIM scan mode, electron energy 70 eV. The concentration series of the fatty acid standard solution were, respectively, detected by GC-MS. Information on standards SCFAs is listed in Table S3. The concentration of the standard was used as the abscissa, and the peak area ratio of the standard and the internal standard was used to measure the linearity of the standard solution. 

### 2.7. RNA Extraction, Library Preparation, Sequencing, and Differential Expression Analysis

Total RNA was extracted from the testis and cecum tissues using a TRIzol reagent kit (Invitrogen, Carlsbad, CA, USA). Only high-quality RNA samples (OD260/280 = 1.8–2.2, OD260/230 ≥ 2.0, RIN ≥ 6.5, 28S:18S ≥ 1.0, >1 μg) were used to construct the sequencing library. Subsequently, the mRNA was enriched using oligo (dT) beads. The enriched mRNA was fragmented and reverse-transcribed into cDNA using random primers. The second-strand cDNA was synthesized using DNA polymerase I. The cDNA fragments were purified, end-repaired, and ligated to the Illumina sequencing adapters. The ligation products were subjected to agarose gel electrophoresis for size selection and sequenced using Illumina HiSeq 4000 (Gene Denovo Biotechnology, Guangzhou, China). To identify differentially expressed genes (DEGs) between the two samples, the expression level of each transcript was calculated according to the fragments per kilobase of exon per million mapped reads (FPKM) method. RSEM (http://deweylab.biostat.wisc.edu/rsem/, accessed on 17 July 2020) was used to quantify gene abundance [41]. Differential expression analysis was performed using DESeq2 based on the cutoffs of *p*-adjust < 0.05 and |log2FC| ≥ 1. Functional enrichment analysis was performed to identify DEGs that were significantly enriched in GO terms and metabolic pathways. Functional enrichment analysis was performed using Goatools (https://github.com/tanghaibao/Goatools, accessed on 17 July 2020). 

### 2.8. Statistical Analysis

Statistical analyses of hormone levels, testicular weight, body weight, and SCFA were performed using one-way analysis of variance (ANOVA) in GraphPad Prism 7. Principal coordinate analysis (PCoA) and Adonis analysis of variance were performed using R version 3.4.5. *p* values were adjusted for multiple comparisons using the Kruskal–Wallis test with the Scheffé post hoc test, and comparisons were evaluated using Welch’s *t*-test. A heat map was constructed using the heatmap2 function in the R gplot package. Multivariate association with linear models (MaAslin) was used for the multivariate assessment of associations between taxa abundance and androgen concentration using default parameters [42]. Redundancy analysis (RDA) was performed using Canoco 5.0 for Windows (V4.5) to ordinate gut microbial abundance to other parameters, including KEGG pathways, SCFA, and androgen levels. 

## 3. Results

### 3.1. Reproductive Performance in Three Groups of Male Plateau Pikas

**Figure 1:**
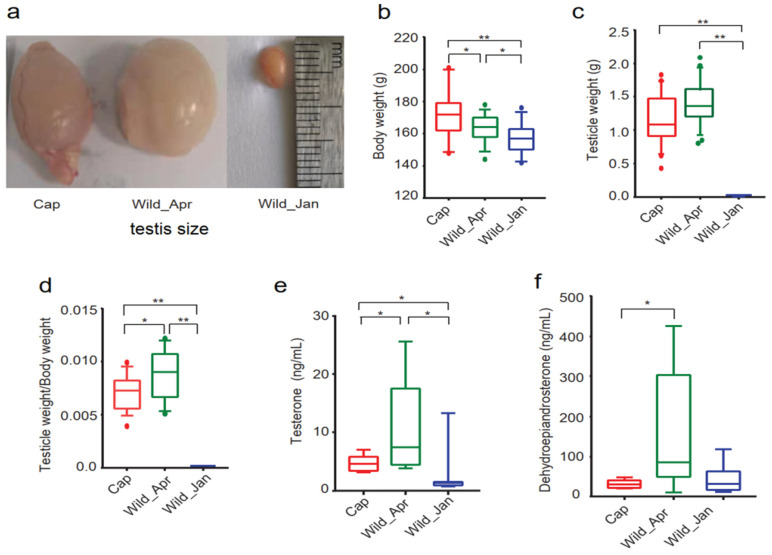
Phenotypic performances in three groups (captive, Cap, *n* = 9; wild pikas caught in April, Wild_Apr, *n* = 12; and wild pikas caught in January, Wild_Jan, *n* = 6). (**a**) Testis size; (**b**) body weight; (**c**) testis weight; (**d**) testis weight/body weight ratio; (**e**,**f**) testosterone and dehydroepiandrosterone concentration of male plateau pikas. Differences were calculated using one-way ANOVA and are denoted as follows: * *p* < 0.05; ** *p* < 0.01.

Plateau pikas undergo seasonal estrus. Seasonal breeding begins in April and ends in July. To determine the differences in reproductive performance between wild and captive plateau pikas, phenotypic data from three groups were collected and compared: wild pikas in January (Wild_Jan), captive pikas (Cap; caught in January and kept in the laboratory until April), and wild pikas in April (Wild_Apr). The testicles of plateau pikas notably increased in April compared with those in January (Figure 1a,c), whereas there was no difference between the captive and wild groups in April (Welch’s *t*-test, Cap vs. Wild_Apr, *p* = 0.0568, Figure 1c). Body weight in the Cap group was significantly higher than that in the wild pika group (Welch’s *t*-test, Wild_Apr vs. Cap, *p* = 0.0382; Wild_Jan vs. Cap, *p* = 0.0052, Figure 1b), whereas for the wild pikas, body weight was higher in April than in January (Welch’s *t*-test, Wild_Jan vs. Wild_Apr, *p* = 0.0393) (Figure 1b). However, the testicle weight/body weight ratio was significantly lower in the captive group than in the wild groups in April (Welch’s *t*-test, Cap vs. Wild_Apr, *p* = 0.0342, Figure 1d). The levels of nine serum hormones—testosterone, dehydroepiandrosterone, estradiol, dopamine, estrone, thyroxine, stanolone, cortisol, and melatonin—were measured in the serum of the three groups. The results revealed that the levels of testosterone (Welch’s *t*-test, Cap vs. Wild_Apr, *p* = 0.0303) and dehydroepiandrosterone (Welch’s *t*-test, Cap vs. Wild_Apr, *p* = 0.0439) were significantly lower in the captive group than in the Wild_Apr (Figure 1e,f and Figure S1). 

**Figure 2:**
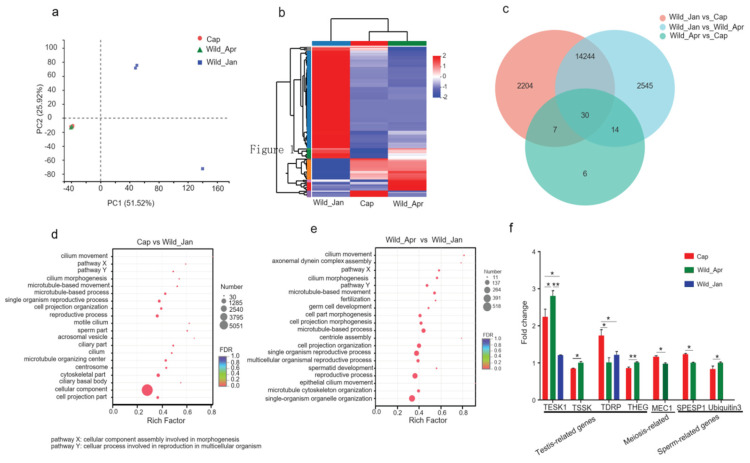
mRNA expression profiles of testicular tissue in three groups (Cap, *n* = 3; Wild_Apr, *n* = 3, Wild_Jan, *n* = 3). (**a**) PCA was performed on differentially expressed genes (DEGs) in the testicular tissue in three groups; (**b**) heat map of differentially expressed genes in three groups; (**c**) Venn diagram showing DEGs among three groups (Cap vs. Wild_Jan, Cap vs. Wild_Apr, and Wild_Jan vs. Wild_Apr); (**d**) GO pathway enrichment analysis of differentially expressed genes between Cap and Wild_Jan groups; (**e**) GO pathway enrichment analysis of differentially expressed genes between Wild_Apr and Wild_Jan groups; (**f**) fold changes in the mRNA expression of testis-related, meiosis-related, and sperm-related genes in the testes were compared among three groups. *p*-values were calculated using one-way ANOVA; * *p* < 0.05, ** *p* < 0.01.

Transcriptome sequencing of the testicles was conducted to explore divergence among the three groups. PCoA based on the expressed genes showed an evident separation between the April and January samples (Figure 2a). In total, 16485 significant DEGs were identified between the Cap and Wild_Jan, with 3381 upregulated and 13104 downregulated genes (Figure S2b); 16833 significant DEGs were identified between Wild_Jan and Wild_Apr, with 13,277 upregulated and 3556 downregulated genes (Figure S2c). However, only 57 significant DEGs were identified between Cap and Wild_Apr, of which 38 were upregulated and 19 were downregulated (Figure S2a). The expression profiles of DEGs were highly divergent (Figure 2b). Thirty genes overlapped between Cap and Wild_Apr, Cap and Wild_Jan, and Wild_Jan and Wild_Apr (Figure 2c), indicating that the shared genes were involved in reproduction. In this experiment, we focused on the changes in reproductive status; therefore, we next analyzed the function of the DEGs in the breeding and no-breeding seasons. The GO enrichment results showed a series of pathways related to the male reproductive process in Cap vs. Wild_Jan groups, including the reproductive process (*p* = 5.06 × 10^−11^), sperm part (*p* = 1.29 × 10^−10^), and centrosome (*p* = 2.95 × 10^−10^) (Figure 2d). In the Cap vs. Wild_Jan groups, the GO enrichment results revealed more pathways related to the male reproduction process, such as fertilization (*p* = 3.05 × 10^−6^), germ cell development (*p* = 3.25 × 10^−6^), centriole assembly (*p* = 5.17 × 10^−6^), spermatid development (*p* = 6.19 × 10^−6^), and reproductive process response (*p* = 6.26 × 10^−6^) (Figure 2e). Furthermore, we investigated the expression of seven selected candidate genes related to the male reproductive pathway, specifically four testis−specific genes (*TESK1*, *TSSK*, *TDRP*, and *THEG*), one meiosis gene (*DMC1*), and two sperm-related genes (*SPESP1* and *GGNBP1*), and found that they were all significantly differentially expressed between the captive and wild-type groups (Figure 2f). 

### 3.2. Composition and Alteration of Microbiota in Three Groups of Male Plateau Pikas

**Figure 3:**
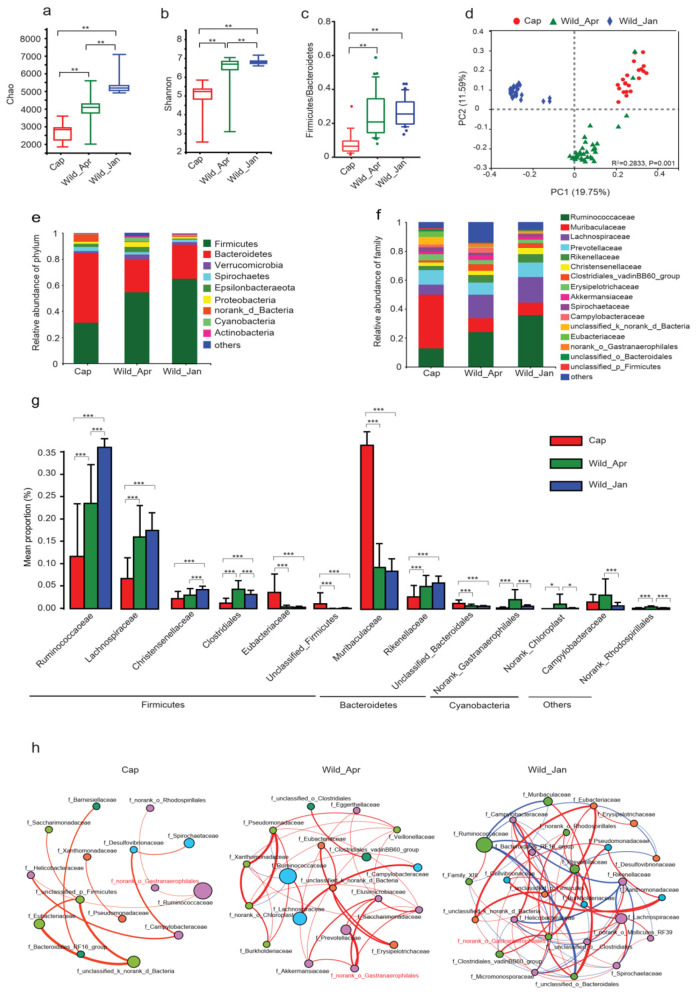
Alteration of the microbial community in three groups (Cap, *n* = 21; Wild_Apr, *n* = 34, Wild_Jan, *n* = 30). (**a**) Chao1 index; (**b**) Shannon index; (**c**) Firmicutes/Bacteroidetes ratio; (**d**) principal coordinate analysis (PCA) based on Bray-Curtis dissimilarity; (**e**) the relative abundances of bacterial communities at the phylum level; (**f**) the relative abundances of bacterial communities at the family level; (**g**) significantly different families in the three groups; (**h**) co-occurrence networks of the top 30 families in three groups. Significance was calculated using the Kruskal–Wallis H test or ANOVA and is denoted as follows: * *p* < 0.05, ** *p* < 0.01, and *** *p* < 0.001. The bacteria of the family emerged in five individuals. Spearman’s correlations > 0.7 or <–0.7 are illustrated, and the line color indicates positive (red) and negative (blue) correlations.

We investigated the fecal microbiota in the captive and wild-type groups. In total, we obtained 10,591,190 16S rDNA sequences from the 85 samples. Subsequently, the sequences were classified into 12,410 OTUs with a 97% identity cutoff. The gut microbial α-diversity indices (Chao, Shannon, and ACE) were drastically decreased in the captivity group based on Welch’s *t*-test (Figure 3a,b and Figure S3a). Furthermore, in wild pika groups, α-diversity was significantly decreased in April compared to that in January. 

At the phylum level, the gut microbiota of the three groups was dominated by *Bacteroidetes* and *Firmicutes* (Figure 3e). The *Firmicutes*/*Bacteroidetes* (F/B) ratio was significantly higher in the wild groups than in the captive group (Welch’s *t*-test, Cap vs. Wild_Apr, *p* < 0.0001; Cap vs. Wild_Jan, *p* < 0.0001, Figure 3c). At the family level, the microbiota of the three groups was dominated by *Ruminococcaceae*, *Muribaculaceae*, *Lachnospiraceae*, and *Prevotellaceae* (Figure 3f). 

Similarities in the bacterial communities among the samples were assessed using Adonis and PCoA based on the Bray–Curtis distance. The PCoA results revealed significant differences in the structure of the gut microbiota among the three groups (Figure 3d, R^2^ = 0.2833, *p* = 0.001). The dissimilarity distances between the captive and wild groups were significantly greater than those between the two wild groups (Figure S3b). 

Discriminatory characteristics were observed among groups based on an average relative abundance of more than 0.1% for OTUs in at least one group. The abundance of *Firmicutes* in the captive group was significantly lower than that in the wild-type group, whereas the abundance of *Bacteroidetes* was two-fold higher in the captive group (Figure S3c). Meanwhile, *Proteobacteria*, *Epsilonbacteraeota*, *Cyanobacteria*, *Tenericutes*, and *Patescibacteria* were abundant in the Wild_Apr group (Figure S3c, Table S4). At the family level, the abundances of *Ruminococcaceae*, *Lachnospiraceae*, *Clostridiales_vadinBB60*, *Rikenellaceae*, *norank_o_Gastranaerophilales*, *norank_o_Chloroplast*, and *norank_o_Rhodospirillales* were significantly lower in the captive group than in the wild group, whereas *Muribaculaceae*, *Eubacteriaceae*, *unclassified_p_Firmicutes*, and *unclassified_o_Bacteroidales* were more abundant in the captive group (Figure 3g, Table S5). Furthermore, *Ruminococcaceae*, *Christensenellaceae*, and *Clostridiales_vadinBB60* in *Firmicutes* were significantly less abundant in the Wild_Apr group than in the Wild_Jan and Cap groups. In addition, the abundance of *Clostridiales_vadinBB60*, *norank_o_Gastranaerophilales*, *norank_o_Chloroplast*, and *norank_o_Rhodospirillales* was significantly higher in the Wild_Apr group (Figure 3g, Table S5). 

Captivity also affects the bacterial network topology. Co-occurrence networks based on the top 30 families revealed the distinct classifications and complexities of the three groups (Figure 3h and Table S5). The simplest network of the Cap group had only 12 links, and almost all the links were positive in the Cap and Wild-Apr groups. In contrast, the network of the Wild_Jan group had a more complicated structure with 65 links, including 38 positive and 27 negative links (Table S6). We found that *f_norank_o_Gastranaerophilales* (highlighted in red in Figure 3h) played a key role, with only positive links in the networks of all three groups. 

### 3.3. Functional Alteration of Microbial Communities in Three Groups of Male Plateau Pikas 

To investigate the differences in the functional capacity of the gut microbiota, we performed metagenomic sequencing of the three groups. Using the KEGG and CAZy databases, we evaluated the functions of the gut microbiota in the three groups. 

**Figure 4:**
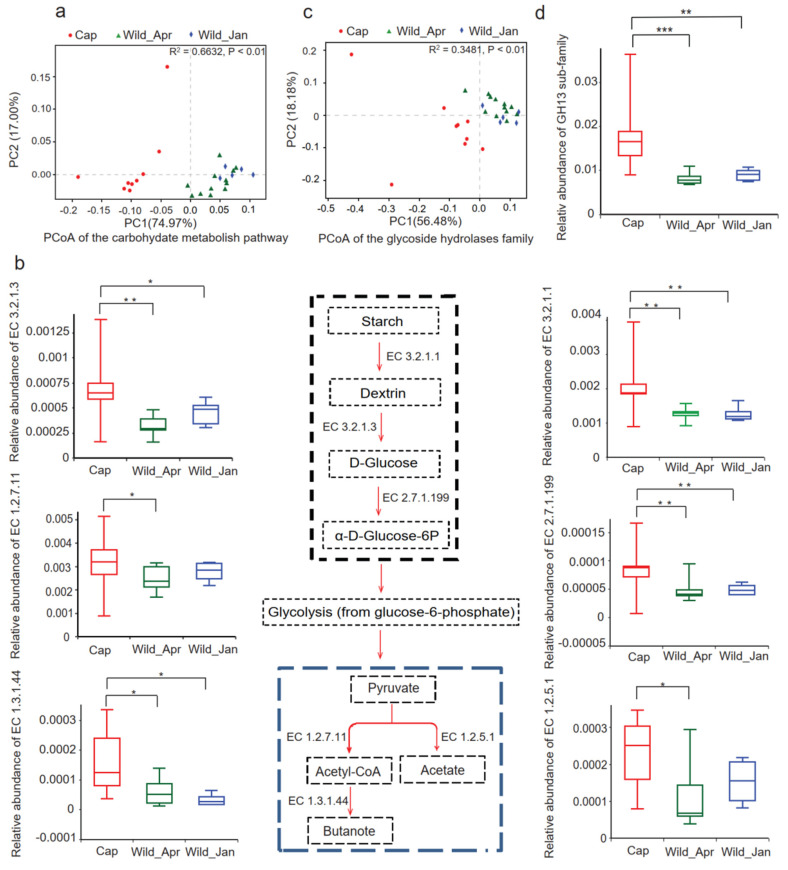
Function alteration in carbohydrate metabolism pathways in three groups. (**a**) PCoA based on the relative abundance of carbohydrate metabolism pathways; (**b**) starch degradation pathway and enzymes were compared among the three groups. Differences were calculated using one-way ANOVA and are denoted as follows: * *p* < 0.05; ** *p* < 0.01, *** *p* < 0.001. A schematic diagram of the pathway for starch degradation is shown in the middle panel; the enzymes involved in starch degradation were compared and listed on both sides; (**c**) PCoA based on the relative abundances of the glycoside hydrolases; (**d**) relative abundance of the GH13 sub-family in three groups.

At the top KEGG level, metabolism was the dominant category (Figure S4a), and carbohydrate metabolism was the primary category at the second KEGG level (Figure S4b). Based on the annotations of the KEGG pathway, PCoA exhibited significant separation, with significant differences between the captive and wild groups (permutations = 999, F = 20.6829, R^2^ = 0.6427, *p* < 0.001, Figure S4c). Significant differences were observed in the carbohydrate metabolite profiles between the captive and wild-type groups (permutations = 999, F = 25.1595, R^2^ = 0.6632, *p* < 0.001; Figure 4a). 

Further analyses revealed that the abundance of enzymes involved in starch and pyruvate metabolism was significantly higher in the captive group than in the wild-type group (Figure 4b). The α-amylase enzyme (EC.3.2.1.1) was highly abundant in the captive group (Kruskal–Wallis H test, corrected *p* = 0.01862), and starch degradation downstream enzymes glucan 1,4-α-glucosidase (EC.3.2.1.3) and D-glucose phosphotransferase (EC.2.7.1.199) were also enhanced in the captive group. Similarly, the key pyruvate metabolism enzymes EC.1.2.7.11 (2-oxoacid oxidoreductase, Kruskal–Wallis H test, corrected *p* = 0.03441) and EC.1.3.1.44 (trans-2-enoyl-CoA reductase, Kruskal–Wallis H test, corrected *p* = 0.02642), together with EC.1.2.5.1 (pyruvate dehydrogenase) for acetate production, were more abundant in the captive group (Figure 4b) than in both wild-type groups. 

In addition, based on the annotations of the CAZy enzyme databases, PCoA exhibited significant differences between the captive and wild groups (permutations = 999, F = 6.5237, R^2^ = 0.36195, *p* < 0.001; Figure S4d). Notably, significant differences were found for glycoside hydrolases between the captive and wild-type groups (permutations = 999, F = 6.1418, R^2^ = 0.3481, *p* < 0.001; Figure 4c). Furthermore, the proportion of the glycosyl hydrolase GH13 family was significantly higher in the captive group than in the wild-type group (Kruskal–Wallis H test, corrected *p* = 0.0066, Figure 4d).

### 3.4. Metabolite Alteration of Microbial Communities in Three Groups of Male Plateau Pikas

**Figure 5:**
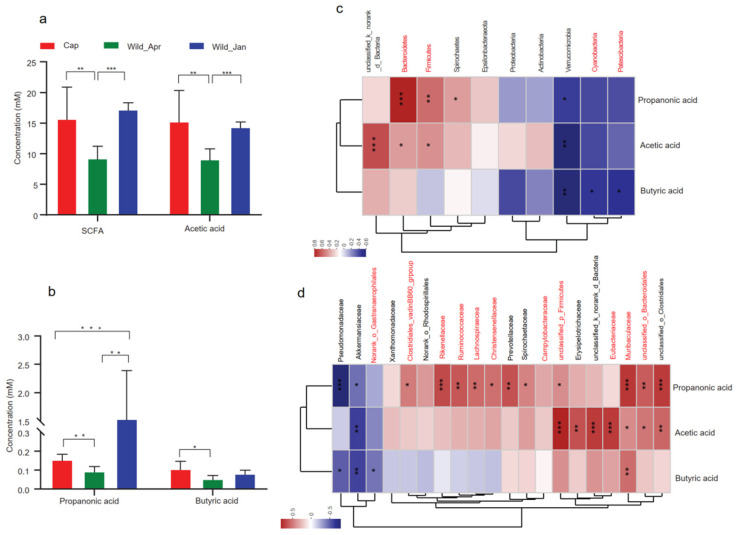
Alteration of short−chain fatty acids (SCFAs) in three groups and the relationship with core bacteria (Cap, *n* = 8; Wild_Apr, *n* = 10, Wild_Jan, *n* = 7). (**a**) Concentration of total SCFAs and acetic acid in the three groups; (**b**) concentrations of propionic and butyric acids in the three groups. *p*-values were calculated using one-way ANOVA and are denoted as follows: * *p* < 0.05, ** *p* < 0.01, *** *p* < 0.001; (**c**) Spearman’s correlation between SCFAs and bacteria (top 30) at the phylum level (the microbe mark in red represents the significant difference in relative abundance at the phylum level in groups); (**d**) Spearman’s correlation between SCFAs and bacteria (top 30) at the family level (the red of the microbe in the hotspot represents the significant difference in relative abundance at the family level in groups).

SCFA concentrations in the three groups were measured. The results showed that SCFA production was higher in the captive group than in the wild-type group (Figure 5a,b). In particular, acetic and butyric acid levels were higher in the captive group (one-way ANOVA, F_2,52_ = 6.653, *p* = 0.0038). Spearman’s correlation between SCFAs and bacteria was also calculated (Figure 5c,d). There was a positive correlation between fecal acetic and propanoic acids and *Bacteroidetes* and *Firmicutes*, both of which were significantly different in abundance between captive and wild groups. A negative correlation was observed between fecal butyric acid and *Cyanobacteria* and *Patescibacteria*, both of which increased significantly in the captive groups (Figure 5c). Furthermore, the acetic acid content showed positive correlations with *Muribaculaceae*, *Eubacteriaceae*, *unclassified_o_Bacteroidales*, and *unclassified_p_Firmicutes*, which were significantly increased in captive groups; butyric acid content showed positive correlations with *Muribaculaceae* and negative correlations with *norank_o_Gastranaerophilales*; and propanonic acid showed positive correlations with *Clostridiales_vadinBB60*, *Rikenellaceae*, *Ruminococcaceae*, *Lachnospiraceae*, *Christensenellaceae*, *unclassified_p_Firmicutes*, *Muribaculaceae*, and *unclassified_o_Bacteroidales* (Figure 5d).

### 3.5. Correlation between Hormones and Microbial Abundance

**Figure 6:**
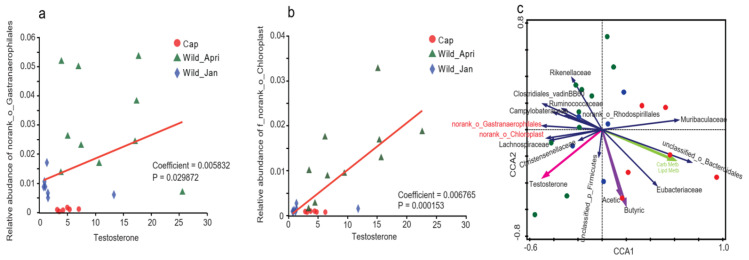
Correlation between androgen and bacteria in three groups. (**a**) Correlations between norank_o_Gastranaerophilales and testosterone concentrations (coefficient = 0.005832, *p* = 0.0299) calculated using MaAsLin; (**b**) correlations between norank_o_Chlorplast and testosterone concentrations (coefficient = 0.006765, *p* = 0.00015) calculated using MaAsLin; (**c**) redundancy analysis (RDA) for the relationships between the dominant bacteria and parameters, including pathways, short-chain fatty acids (SCFAs), and androgens. RDA was performed using Canoco 5.0 for Windows (V4.5). Only the taxa with a mean relative abundance of more than 1% were selected, and dominant bacteria (dark blue arrows), pathways (green arrows), SCFAs (purple arrows), and androgens (red arrows) are shown.

Associations between bacterial communities and androgens (testosterone and dehydroepiandrosterone) were analyzed using multivariate association with linear models (MaAsLin). The results showed that the relative abundances of 28 bacterial families were significantly associated with testosterone levels. Specifically, the abundances of 26 families were positively correlated with testosterone levels, whereas those of two families were negatively correlated with testosterone levels (Table S7, MaAsLin, *p* < 0.05). Significant positive correlations were detected between testosterone and *f_norank_o_Gastranaerophilales* (Figure 6a; MaAsLin, coefficient = 0.0058, *p* = 0.0299) and *f_norank_o_Chloroplast* (Figure 6b; MaAsLin, coefficient = 0.0068, *p* = 0.0002), both of which belong to the phylum *Cyanobacteria*. Significant correlations were also detected between testosterone levels and the relative abundance of the phylum *Cyanobacteria* (Figure S5a; MaAsLin coefficient = 0.0082; *p* = 0.0073). In addition, the relative abundances of two bacterial families (*Vibrionaceae* and *Leuconostocaceae*) were significantly associated with dehydroepiandrosterone levels (Table S8). 

Spearman’s correlations between testosterone, dehydroepiandrosterone, and bacteria were calculated. A negative correlation existed between testosterone levels and the abundance of *Bacteroidetes*, which increased significantly in the Cap group (Figure S5b). Furthermore, testosterone levels were negatively correlated with the abundances of *Muribaculaceae*, *Rikenellaceae*, *unclassified_o_Bacteroidales*, and *clostridiales_vadinBB60_groups*. The abundances of *Muribaculaceae* and *unclassified_o_Bacteroidales* were high in the Cap groups, whereas those of *Rikenellaceae* and *clostridiales_vadinBB60_groups* were high in the wild-type groups (Figure S5c). Meanwhile, *f_norank_o_Gastranaerophilales* were positive for testosterone levels, which is consistent with the results of MaAsLin. In contrast, there was no significant correlation between the abundance of bacteria and dehydroepiandrosterone levels (Figure S5b,c).

To better show the relationship between flora, metabolites, and host, the canonical correspondence analysis (CCA) was conducted to investigate the correlation between bacterial abundance and other factors, including SCFAs, androgens, and KEGG pathways. The microbial community structure was shaped by several primary factors, including nine hormones, SCFAs and five targeted KEGG pathways. After removing redundant variables, five factors, namely testosterone, acetic acid, butyric acid, and the pathways of carbohydrate and lipid metabolism (Carb metb and Lipd Metb), were selected for further redundancy analysis (Figure 6c). The results revealed several significant correlations between the bacteria and these factors. For example, *Muribaculaceae* and *unclassified_o_Bacteroidales* were positively correlated with lipid and carbohydrate metabolism pathways; *Eubacteriaceae* and *unclassified_p_Firmicutes* were positively correlated with acetate, butyrate, and lipid and carbohydrate pathways; *norank_o_Chloroplast* and *norank_o_Gastranaerophilales* were positively correlated with testosterone. The correlation between microbial, testosterone, and SCFA levels is consistent with the results of previous studies.

### 3.6. Expression Profiles Changes in Cecum Male Plateau Pika Epithelium Tissue

**Figure 7:**
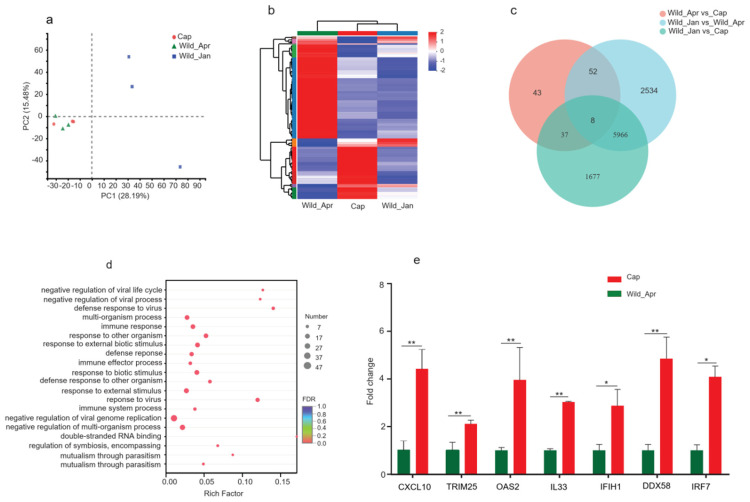
mRNA expression profiles of cecum epithelial tissue in three groups (Cap, *n* = 3; Wild_Apr, *n* = 3, Wild_Jan, *n* = 3). (**a**) Principal component analysis (PCA) was performed on differentially expressed genes (DEGs) in the cecum epithelial tissue in the three groups; (**b**) heat map of DEGs in three groups; (**c**) Venn diagram showing DEGs among three comparisons (Cap vs. Wild_Jan, Cap vs. Wild_Apr, and Wild_Jan vs. Wild_Apr; (**d**) GO pathway enrichment analysis of DEGs between Cap and Wild_Apr groups; (**e**) fold changes in the mRNA expression of immune-related genes in cecum epithelial tissues were compared between Cap and Wild_Apr groups. *p*-values were calculated using one-way ANOVA, * *p* < 0.05, ** *p* < 0.01.

We performed transcriptome sequencing of the cecum epithelial tissue to investigate the effects of the microbiota on gene expression in the epithelium. Gene expression profiles were identified in the cecum epithelium and were evidently separated among the groups (Figure 7a). In total, 7688 significant DEGs were identified between Cap and Wild_Jan groups, with 4096 upregulated and 3592 downregulated genes (Figure S6a); 8560 significant DEGs were identified between Wild_Jan and Wild_Apr groups, with 4265 upregulated and 4295 downregulated genes (Figure S6c). However, only 140 significant DEGs were identified between Cap and Wild_Apr groups, of which 88 were upregulated and 52 were downregulated (Figure S6b). The expression profiles of DEGs were highly divergent (Figure 7b). Eight genes overlapped between Cap and Wild_Apr, Cap and Wild_Jan, and Wild_Jan and Wild_Apr groups, indicating that shared genes were involved in response to changes at different time points. In this experiment, we focused on the effects of captivity; therefore, we analyzed the functions of the DEGs in Cap and Wild_Apr groups. GO enrichment analysis showed that the immune response (*p* = 2.36 × 10^−12^), immune effects process (*p* = 3.68 × 10^−11^), and immune system process (*p* = 1.18 × 10^−10^) pathways were enriched and related to host immunity (Figure 7d). In particular, the expression of several pro-inflammatory cytokines, especially seven DEGs (C-X-C motif chemokine (*CXCL*)*-10*, *TRIM25*, *OAS2*, interleukin (IL)-33 (*IL33*), *IFIH1*, *DDX58*, and *IRF7*), was significantly elevated in the epithelia of the captive group (Figure 7e).

## 4. Discussion

### 4.1. Captive Breeding Impairs Male Fecundity

Wild animals brought into captivity must cope with a series of drastic changes imposed by artificial conditions, such as living space, food conditions, and community relationships. These can cause endocrine disorders [43], obesity [8], decreased immune levels [9], and infertility [10]. We found that plateau pikas also faced an unparalleled crisis in captivity.

Plateau pikas are seasonally breeding small herbivores. In this study, the weight of adult pikas’ testes, testosterone levels, and the testicle weight/body weight ratio increased significantly in breeding periods (both in captivity and wild) compared with no-breeding periods. It indicated that in captivity, the plateau pikas still obeyed the rules of seasonal breeding. However, captive feeding increased body weight, but reduced testosterone levels and the testicle weight/body weight ratio in the breeding periods. These majorly contributed to infertility in captivity. Deficiency in testosterone production was a major cause of male reproductive disorders [44,45]. High-calorie diets can increase body weight, lead to severe endocrine dysfunction, and ultimately induce male subfertility [46]. In this study, nitrogen-free extracts (mainly starch) were the major constituents of artificial feed, which could provide sufficient energy for animals (Table S2). Several studies, including those on humans, birds, and mammals in captivity, have revealed that a high-energy diet increases body weight and is detrimental to male fertility [47,48,49]. Excessive energy diet-induced metabolic syndrome (MetS) results in abnormal testicular tubules and a sharp decline in spermatogonia differentiation [50]. The gene expression profiles of testes tissue also revealed significant differences in breeding periods compared with no-breeding periods. It implied that the captive plateau pikas definitely entered the mating condition. However, the gene expression profiles between captivity and wild were indeed different in mating season between captivity and wild in mating season. Significant reductions were detected in the male reproductive-related gene, including testis-specific kinase 1 (*TESK1*), testis-specific serine kinase (*TSSK*), testis development-related protein (*TDRP*), testicular haploid expressed gene (*THEG*); meiotic recombination protein (*DMC1*), sperm equatorial segment protein 1 (*SPESP1*); and gametogenetin-binding protein 1-like (*GGNBP1*) in the captive groups. *TESK1* is important in meiosis [51]. Reduction in *Tssk* causes a significant decrease in the fertilization ability of male mice [52]. *TDRP1* is expressed in spermatogenic cells and is associated with spermatogenesis and reproductive traits in mammals [53]. *Theg*, predominantly expressed in the nuclei of spermatids, is associated with weight reduction in the testes [54]. *SPESP1* is involved in sperm–egg binding and fusion [55]. Previous evidence suggests persistent energy excess has an impact on fertility, and men with obesity frequently display low testosterone levels [56,57]. The abnormal expression of these genes may be one cause of testosterone defects and low testicle weight/body weight ratios. 

### 4.2. Captivity Alters Gut Microbiome 

Entrance to captivity is commonly accompanied by gut microbiota dysbiosis [5]. α−Diversity was significantly lower in captivity than in wild animal populations. This pattern is consistent with results observed in humans and other animals. Studies on amphibians [58,59], birds [60], and mammals [61,62] have shown that microbial diversity is reduced in captive hosts. Consistent with these results, we found a significant reduction in α-diversity in captive plateau pikas. This discrepancy may be attributed to two reasons. The first is artificial food, as artificial diets for captive animals contain high levels of nutrients, including starch, protein, and fat, resulting in a significant reduction in gut microbial diversity in captive animals. Second, captive breeding blocked contact between animals and horizontal transmission of bacterial communities. In nature, a certain overlap in food, space, and other resources among sympatric animals increases the horizontal transmission of microbes in the host [63,64]. In addition, plateau pikas engage in coprophagy, and this behavior allows mammals to recover nutrients and stabilize the gut microbiome via horizontal transmission in wild mammals [65]. 

*Firmicutes* dominated the gut microbiome of wild pikas, whereas *Bacteroidetes* was the dominant phylum in captive pikas; this profile was also observed in deer mice [66], primates [67], and pangolins [58]. The change in the F/B ratio indicated dysbiosis of gut microbes, and the same profiles of microbes were also observed in males with excessive energy diet-induced MetS and HFD [68]. An increased F/B ratio can improve energy acquisition in food because *Firmicutes* are associated with digestion efficiency and energy harvesting [69]. In the *Firmicutes* phylum, *Ruminococcaceae*, *Lachnospiraceae*, and *Clostridiales* play important roles as degraders of complex plant materials [70], and *Christensenellales* are involved in the metabolic conversion of nondigestible carbohydrates [71]. The natural habitat of plateau pikas is the Qinghai–Tibet Plateau, and food shortages are a severe challenge for survival in this region. Highly cellulolytic bacteria are essential for the microbial breakdown of cellulose in plateau pikas during a famine. In comparison, the increased abundance of *Bacteroides* may be associated with the dietary starch in captive pikas. *Bacteroides* can degrade carbohydrates (especially polysaccharides), proteins, and other substances to increase the nutrient utilization rate of the host [72]. Further analysis revealed that abundant *Bacteroides*, mainly caused by an increase in the *Muribaculaceae* family, which is usually found in the rodent gut, can degrade various complex carbohydrates and is capable of utilizing lactate and converting it to acetate [73]. Furthermore, the networks of the main bacteria differed among the groups. The network of the wild-type group was much more complicated than that of the Cap group. In metabolite-rich environments, bacteria can select for the loss of biosynthetic genes, thus resulting in oversimplification of the network, whereas in metabolite-poor environments, the local exchange among cooperative bacteria and reciprocity increases [74]. The alteration in microbes indicates that intestinal microorganisms can quickly adapt to captive breeding [75].

A perturbed (dysbiosis) microbiota may be followed by a disrupted function. Metagenome assembly showed that the alteration of microbes also modified their function (Figure 4 and Figure S4). Artificial feeding contains high levels of starch, and the substantial differences may drive the increase in microbiota carbohydrate degradation. The metabolic functions of the captive microbiome mapped well onto artificial foods. In the present study, the captive microbiome was enriched with enzymes involved in starch metabolism and carbohydrate degradation. Captive pangolins and musk deer also showed an increased capacity for carbohydrate metabolism [58,76]. The abundant starch content in artificial foods necessitates the presence of pathways for the fermentation and degradation of starch. Starch metabolism is implicated in the production of SCFAs, acetate, propionate, butyrate, and especially acetic acid. Acetate production and energy storage increase when the gut microbiota is exposed to calorically dense nutrients [46]. A murine model study showed that high fecal SCFA concentrations were positively associated with body weight and increased with a calorie-rich diet [46]. In contrast, a stable and functional equilibrium in the microbiome was observed in both wild groups. Functional redundancy is necessary to ensure the stability of the microbiota [77] and that they are resistant to chaotic blooms of subpopulations [72].

### 4.3. Dysbiosis of Microbe in Captivity Exacerbates Male Infertility in Plateau Pika

Human studies have demonstrated that pregnancy shapes the intestinal microbiota, prompting metabolic changes that may favor fertility and reproduction [78,79]. The gut microbiome during pregnancy and postpartum is significantly altered in the eastern black rhino [15]. In our study, we also demonstrated that gut microbial communities change concurrently with reproductive status in wild male plateau pikas, and the gut microbiomes during estrus and anestrus are significantly different. However, the profile of microbes in captivity is different from that of the two periods, while the microbiota structure is closer to that of April; this implies that in captivity, the microbe also changes with the reproduction status, or in part, linked to the physiological status. Androgen deprivation alters the composition of fecal microbiota in a high-fat diet [80]. Microbial communities can also alter sex hormone levels [81]. Our results revealed that the abundances of *norank_o_Gastranaerophilales* and *Cyanobacteria* were positively correlated with testosterone levels, which may be associated with androgen synthesis, as both of them possess 7α-hydroxysteriod dehydrogenases (7α-HSDs) [82], which are key enzymes in the metabolism of steroids in prokaryotes and eukaryotes [83]. The abundance of chloroplasts also changed with testosterone levels. We speculate that this association is related to food; plateau pikas can obtain green plants as food in the estrous stage in the wild. The chloroplast content is substantial in wild populations but decreases or is nearly absent in semi-captive and captive primate gorilla populations [67]. 

In addition to androgens, profile changes in captive microbes cause alterations in product metabolism. Increased SCFA production causes acidosis [84] and intestinal inflammation [85]. In addition, SCFAs influence immune responses via immune-related gene expression [86]. In our study, we observed significantly high expression of immune-related genes, including *DDX58*, *IRF7*, *IL-33*, *IFIH1*, *CXCL-10*, and *OAS2*, in the cecal tissue of captive pikas. *IL-33* is a member of the IL-1 family [13], which increases in the caput epididymal cells of high-fat-diet-fed mice [87]. Furthermore, *CXCL10* expression is increased in the caput epididymal cells of HFD-fed mice and roosters [13,88]. *DDX58*, *IFIH1*, *IRF7*, and *OAS2* are essential in the inflammatory response [89,90,91]. Immune-related genes are critical for immunity and are involved in the impairment of testicular function [92]. Thus, in captivity, dysbiosis of the gut microbiota affects the host testicles via systemically elevated inflammatory responses [49].

## 5. Conclusions

Our study provides valuable information on the captive breeding of male pikas and elucidates the importance of microbes in captive reproduction, which poses great potential for the conservation of endangered species. In the future, our results would benefit the application of microbiome in endangered species, to probe host health, nutrition, and disease in a non-invasive way.

## Data Availability

The 16S sequencing data, metagenomic data, and RNA-Seq data have been deposited in the Sequence Read Archive (SRA, https://www.ncbi.nlm.nih.gov/sra, accessed on 5 January 2023) of NCBI, with accession No. PRJNA774646 (16s), PRJNA777737 (metagenome), and PRJNA810901 (mRNA).

## Supplementary Materials

The following supporting information can be downloaded at https://www.mdpi.com/article/10.3390/biom14040403/s1, Figure S1: Hormone concentration of male plateau pikas. Figure S2: Volcano plots showing the changes in genes in testicular tissue between Cap and Wild_Jan, Cap and Wild_Apr, and Wild_Jan and Wild.Apr groups. Figure S3: Alteration of the α, β diversity and composition of microbial community in three groups. Figure S4: Function alteration in KEEG pathways and CAZymes in three groups. Figure S5: Correlation between androgen and bacteria in phylum and family level. Figure S6: Volcano plots showing the changes in genes in cecum epithelial tissue between Cap and Wild_Jan, Cap and Wild_Apr, and Wild_Jan and Wild_Apr groups. Table S1: Contents of the artificial and wild diets. Table S2: Samples and investigations conducted in this study. Table S3: The information on animal hormone and SCFA standards in this study. Table S4: Results of the Wilcoxon rank-sum test comparing the abundance among groups in the phylum. Table S5: Results of the Wilcoxon rank-sum test comparing the abundance among groups in families. Table S6: Network and topological indices of the top 30 bacteria in the co-occurrence networks. Table S7: Association analysis results between testosterone and bacterial families using the multivariate association with linear models (MaAsLin) method. Table S8: Association analysis results between dehydroepiandrosterone and bacterial families using the MaAsL method.
