# Supplementary material for: Dysbiosis of Gut Microbiome Aggravated Male Infertility in Captivity of Plateau Pika
Source: Biomolecules. 2024 Mar 26;14(4):403. doi: 10.3390/biom14040403 (PMC11047922; doi:10.3390/biom14040403)
Supplement: Supplementary file 1 [file biomolecules-14-00403-s001.zip › Fig_S1_S6.pdf]

Figure. S1

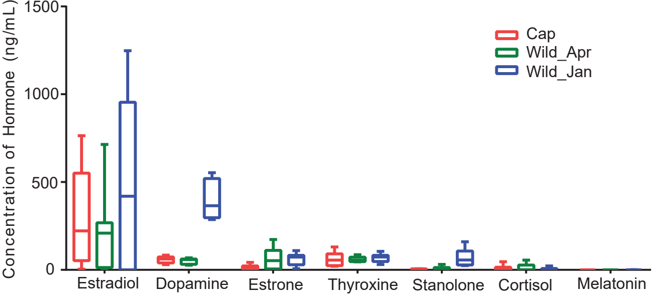

1500
1000
500
0
Concentration of Hormone (ng/mL)
Cap
Wild_Apr
Wild_Jan
Estradiol
Dopamine
Estrone
Thyroxine
Stanolone
Cortisol
Melatonin

Figure. S2

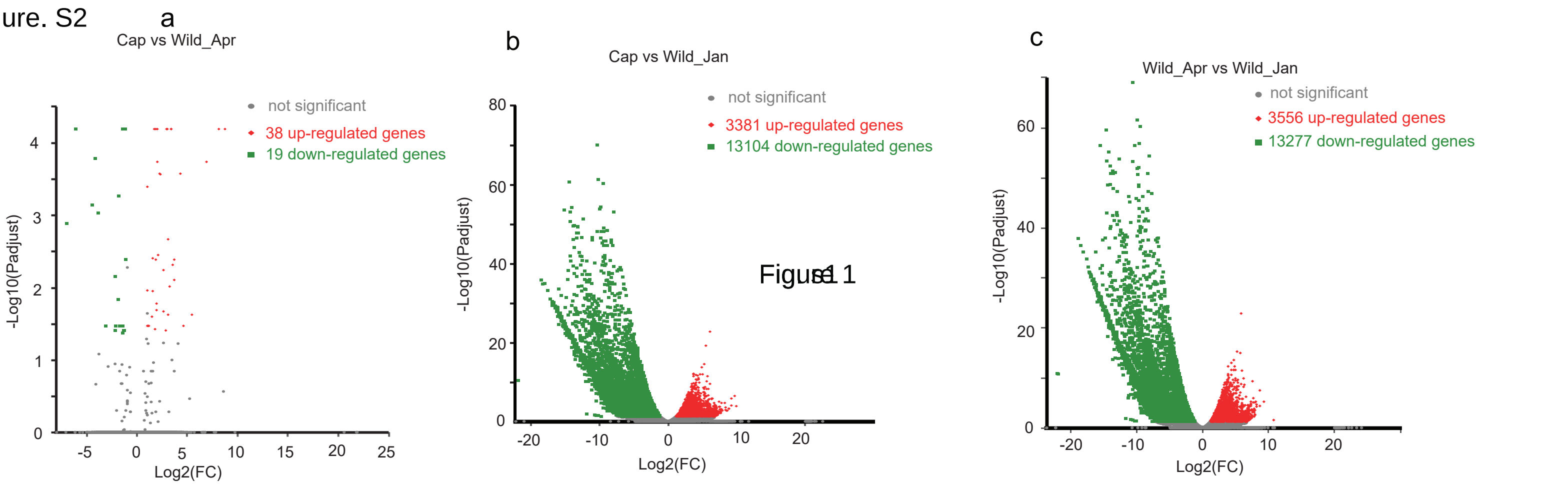
a
Cap vs Wild_Apr
not significant
38 up-regulated genes
19 down-regulated genes
-Log10(Padjust)
Log2(FC)
b
Cap vs Wild_Jan
not significant
3381 up-regulated genes
13104 down-regulated genes
-Log10(Padjust)
Log2(FC)
c
Wild_Apr vs Wild_Jan
not significant
3556 up-regulated genes
13277 down-regulated genes
-Log10(Padjust)
Log2(FC)

Figure. S3

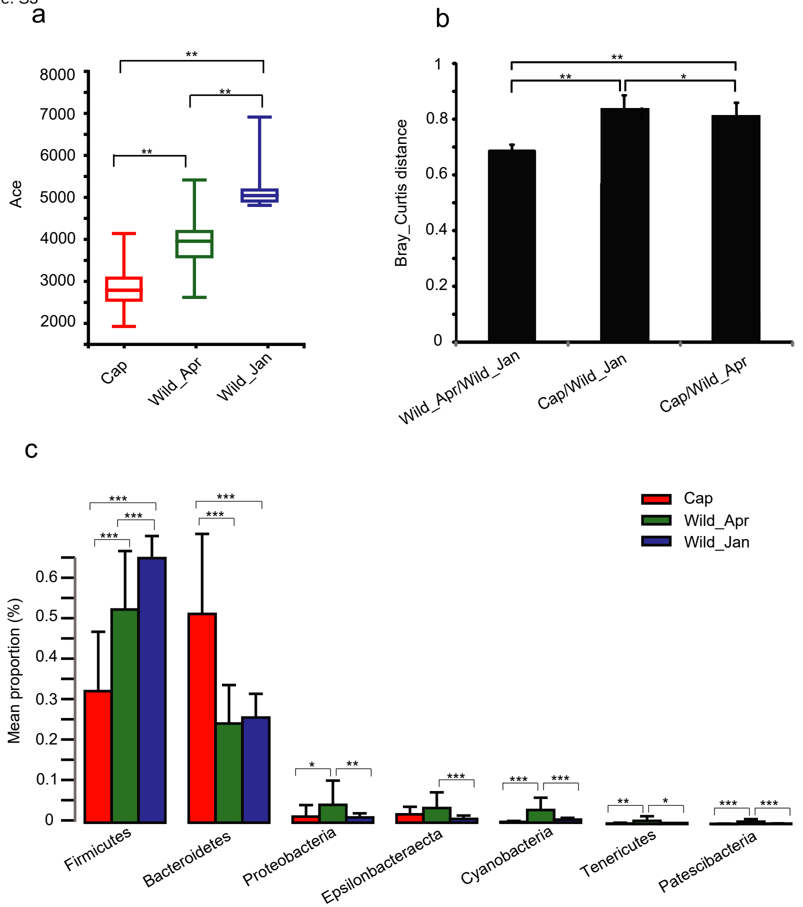
a
8000
7000
6000
5000
4000
3000
2000
Ace
**
**
**
Cap
Wild_Apr
Wild_Jan
b
1
0.8
0.6
0.4
0.2
0
Bray_Curtis distance
**
**
*
Wild_Apr/Wild_Jan
Cap/Wild_Jan
Cap/Wild_Apr
c
Cap
Wild_Apr
Wild_Jan
0.6
0.5
0.4
0.3
0.2
0.1
0
Mean proportion (%)
***
***
***
*
**
**
*
Firmicutes
Bacteroidetes
Proteobacteria
Epsilonbacteraecta
Cyanobacteria
Tenericutes
Patescibacteria

Figure. S4

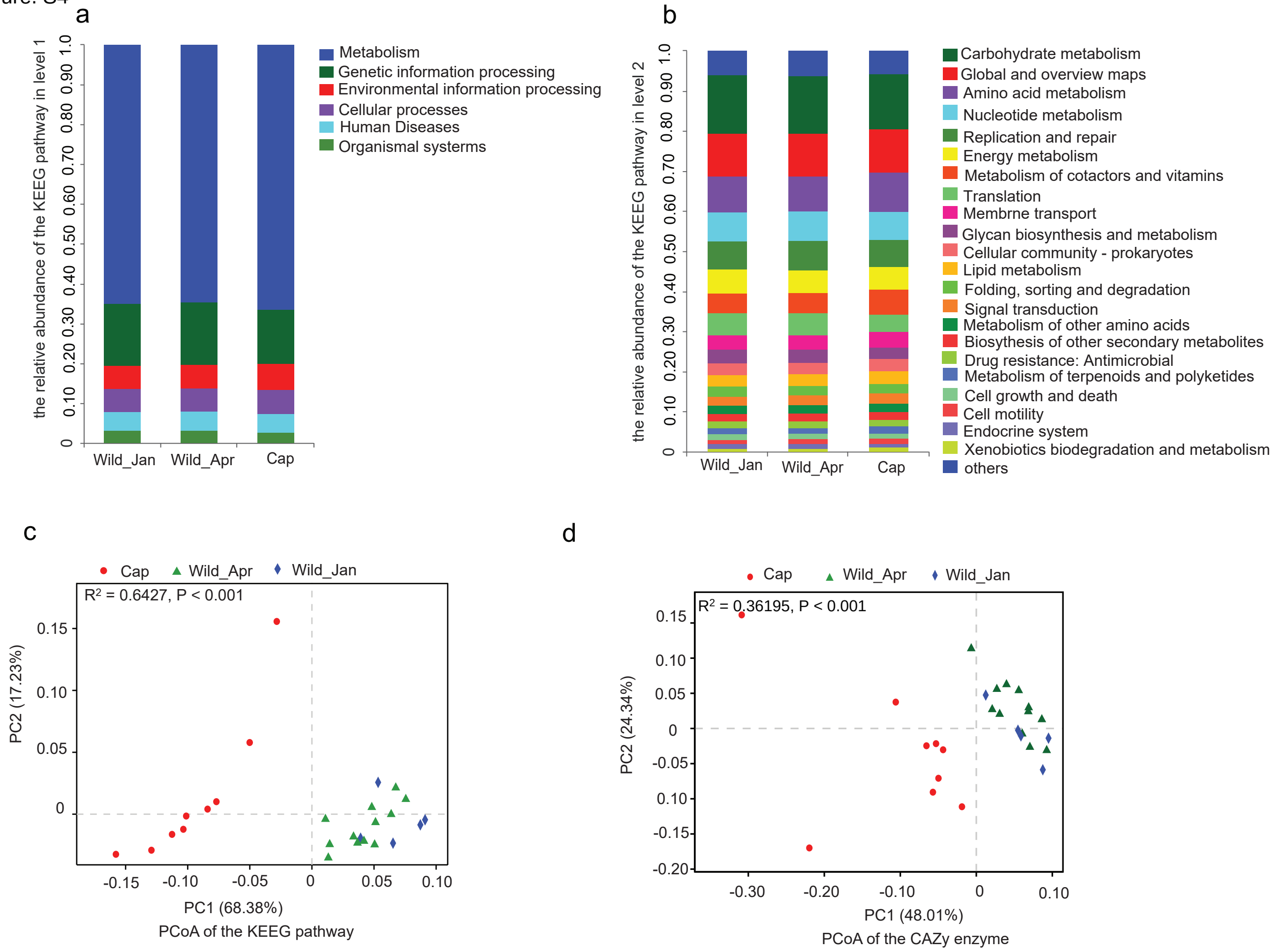
a
the relative abundance of the KEEG pathway in level 1
0
0.10
0.20
0.30
0.40
0.50
0.60
0.70
0.80
0.90
1.0
Wild_Jan
Wild_Apr
Cap
Metabolism
Genetic information processing
Environmental information processing
Cellular processes
Human Diseases
Organismal systerms
b
the relative abundance of the KEEG pathway in level 2
0
0.10
0.20
0.30
0.40
0.50
0.60
0.70
0.80
0.90
1.0
Wild_Jan
Wild_Apr
Cap
Carbohydrate metabolism
Global and overview maps
Amino acid metabolism
Nucleotide metabolism
Replication and repair
Energy metabolism
Metabolism of cotactors and vitamins
Translation
Membrne transport
Glycan biosynthesis and metabolism
Cellular community - prokaryotes
Lipid metabolism
Folding, sorting and degradation
Signal transduction
Metabolism of other amino acids
Biosythesis of other secondary metabolites
Drug resistance: Antimicrobial
Metabolism of terpenoids and polyketides
Cell growth and death
Cell motility
Endocrine system
Xenobiotics biodegradation and metabolism
others
c
Cap
Wild_Apr
Wild_Jan
R² = 0.6427, P < 0.001
PC2 (17.23%)
0.15
0.10
0.05
0
-0.15
-0.10
-0.05
0
0.05
0.10
PC1 (68.38%)
PCoA of the KEEG pathway
d
Cap
Wild_Apr
Wild_Jan
R² = 0.36195, P < 0.001
PC2 (24.34%)
0.15
0.10
0.05
0
-0.05
0.10
-0.15
-0.20
-0.30
-0.20
-0.10
0
0.10
PC1 (48.01%)
PCoA of the CAZy enzyme

Figure. S5

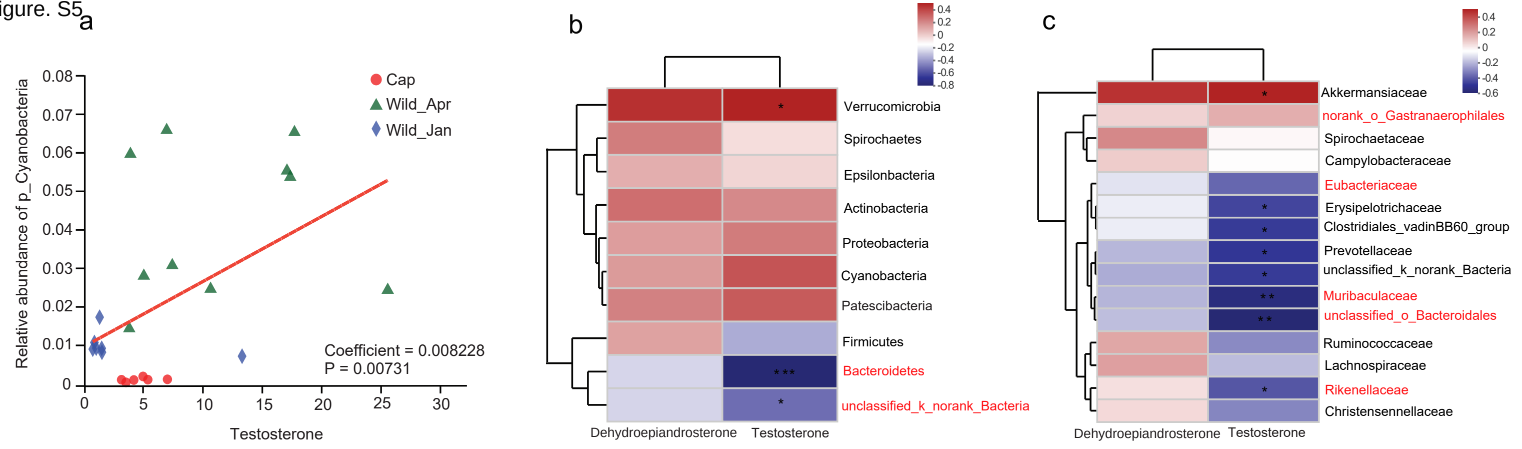
a
Relative abundance of p_Cyanobacteria
0.08
0.07
0.06
0.05
0.04
0.03
0.02
0.01
0
0
5
10
15
20
25
30
Testosterone
Cap
Wild_Apr
Wild_Jan
Coefficient = 0.008228
P = 0.00731
b
0.4
0.2
0
-0.2
-0.4
-0.6
-0.8
Verrucomicrobia
Spirochaetes
Epsilonbacteria
Actinobacteria
Proteobacteria
Cyanobacteria
Patescibacteria
Firmicutes
Bacteroidetes
unclassified_k_norank_Bacteria
Dehydroepiandrosterone
Testosterone
c
0.4
0.2
0
-0.2
-0.4
-0.6
Akkermansiaceae
norank_o_Gastranaerophilales
Spirochaetaceae
Campylobacteraceae
Eubacteriaceae
Erysipelotrichaceae
Clostridiales_vadinBB60_group
Prevotellaceae
unclassified_k_norank_Bacteria
Muribaculaceae
unclassified_o_Bacteroidales
Ruminococcaceae
Lachnospiraceae
Rikenellaceae
Christensenellaceae
Dehydroepiandrosterone
Testosterone

Figure. S6

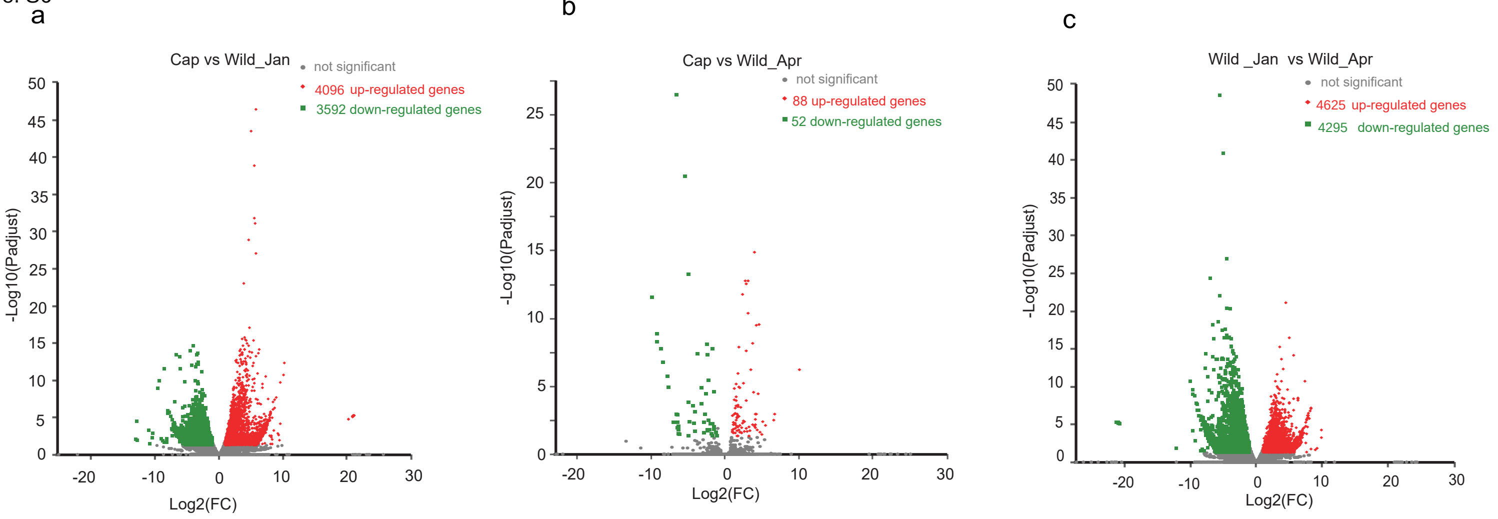
a
Cap vs Wild_Jan
not significant
4096 up-regulated genes
3592 down-regulated genes
-Log10(Padjust)
Log2(FC)
b
Cap vs Wild_Apr
not significant
88 up-regulated genes
52 down-regulated genes
-Log10(Padjust)
Log2(FC)
c
Wild _Jan vs Wild_Apr
not significant
4625 up-regulated genes
4295 down-regulated genes
-Log10(Padjust)
Log2(FC)
